# Dynamic Non-Covalent Exchange Intrinsic Self-Healing at 20 °C Mechanism of Polyurethane Induced by Interactions among Polycarbonate Soft Segments

**DOI:** 10.3390/polym16070924

**Published:** 2024-03-27

**Authors:** Yuliet Paez-Amieva, José Miguel Martín-Martínez

**Affiliations:** Adhesion and Adhesives Laboratory, University of Alicante, 03080 Alicante, Spain; yuliet.paez@ua.es

**Keywords:** polyurethane, polycarbonate diol polyol, intrinsic self-healing, soft segments, carbonate–carbonate interactions, mechanism of self-healing

## Abstract

Two polyurethanes (PUs) were similarly synthesized by reacting a cycloaliphatic isocyanate with 1,4-butanediol and two polyols of different nature (polyester, polycarbonate diol) with molecular weights of 1000 Da. Only the PU synthesized with polycarbonate diol polyol (YCD) showed intrinsic self-healing at 20 °C. For assessing the mechanism of intrinsic self-healing of YCD, a structural characterization by molecular weights determination, infrared and X-ray photoelectronic spectroscopies, differential scanning calorimetry, X-ray diffraction, thermal gravimetric analysis, and dynamic mechanical thermal analysis was carried out. The experimental evidence concluded that the self-healing at 20 °C of YCD was due to dynamic non-covalent exchange interactions among the polycarbonate soft segments. Therefore, the chemical nature of the polyol played a key role in developing PUs with intrinsic self-healing at 20 °C.

## 1. Introduction

**Scheme 1:**
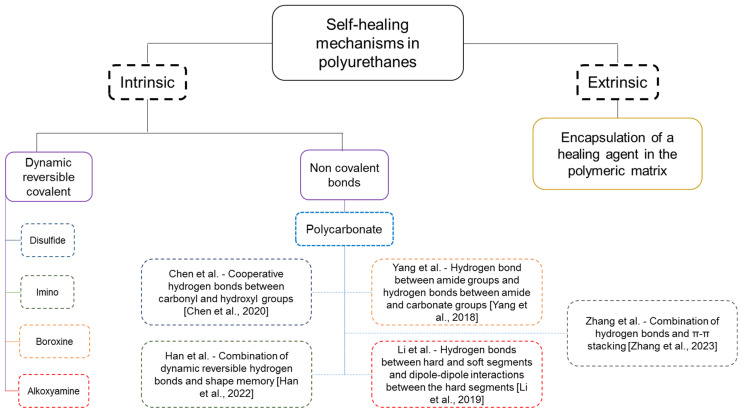
Overview of different mechanisms of self-healing in PUs [71,72,73,74,76].

There is a current interest in developing self-healing polymeric materials for innovative applications in biomedical devices [1], construction materials, the aerospace industry, and electronics [2]. Self-healing can be produced by an extrinsic (encapsulation of a healing agent in the polymeric matrix) or an intrinsic (dynamic reversible covalent or non-covalent bonds) mechanism [3,4,5,6,7,8]—Scheme 1. Intrinsic self-healing in different polymers has been ascribed to the existence of inherent reversible bonds of different nature produced by Diels–Alder (DA) reactions [9,10,11,12], cycloaddition [13], acylhydrazone bonds [14,15,16,17], trithiocarbonate bonds [18,19,20], disulfide bonds [21,22,23,24,25], diarylfuranone bonds [26], hydrogen bonding [27,28], hydrophobic interactions [29,30,31,32], π–π stacking [33,34,35], metal–ligand interactions [36,37,38,39], and ionic interactions [40,41,42,43]. The majority of self-healing polymers need heating at 80–120 °C, and, in general, they show reduced mechanical properties [9,44].

Polyurethanes (PUs) have inherent dynamic bonds, i.e., hydrogen bond interactions between urethane and/or urea groups [45]. PUs are obtained by addition reactions of polyols and polyisocyanates [46,47]. The structure of the most PUs consist of alternating “soft” segments made of polyol with a low glass transition temperature (T_g_) and “hard” segments made by reacting isocyanates with short-chain diols. The difference in polarity and chemical nature between the hard and soft segments leads to micro-phase separation in PUs, which imparts molecular mobility in some regions (soft phase) and physical and structural integrity (hard phase) in others [48,49,50,51].

The self-healing in polymers involves two consecutive stages, i.e., cloture followed by healing, similar to biological healing of skin wounds [52]. These stages require dynamic links, in addition to mobility of the polymer chains [53,54,55]. Some self-healing PUs exhibit shape memory due to physical crosslinking (polar interactions, hydrogen bonds), crystallization of the hard segments, and molecular motion of the soft segments [56]. Xu et al. have designed shape memory-assisted self-healing PUs made with polytetramethylene glycol and epsilon-caprolactone diol; they contained disulfide bonds [57,58,59]. In a different approach, other researchers have reported the synthesis of low-molecular-weight PUs with end groups that can be assembled by hydrogen bonding and π–π stacking interactions to produce thermally reversible supramolecular networks with weak non-covalent interactions [60,61,62,63]. Feula et al. synthesized a supramolecular PU elastomer that self-healed at 45 °C, which was caused by hydrogen bonds and aromatic π–π stacking [64].

Intrinsic self-healing PUs can be produced by dynamic reversible covalent or non-covalent bonds. Self-healing PUs based on dynamic reversible covalent bonds exhibit high binding energy that ensures adequate mechanical strength. The most common dynamic reversible covalent bonds in self-healing PUs are disulfide, diselenide, ditelenide, imino, boroxine, and alkoxyamine [65]—Scheme 1. Aliphatic and aromatic disulfide bonds are common in room-temperature self-healing PUs, which are induced by ultraviolet light stimulation, and they show a yellow appearance and low transparency [66]. Jing et al. have obtained PUs with aromatic disulfide bonds and an important number of hydrogen bonds, and a relatively quick self-healing (5 h at 60 °C or 24 h at room temperature) combined with a high tensile strength (8.0 MPa) were reported [67]. On the other hand, the combination of dual or triple dynamic reversible covalent bonding in PUs may assure stronger and rapid self-healing. Thus, Xu et al. [68] have synthesized a room-temperature self-healing PU that contains multiple hydrogen bonds and zinc–imidazole coordination bonds. The multiple weaker hydrogen bonds dissipated energy effectively and improved the toughness, while the stronger coordination bonds maintained the integrity of the PU structure.

Intrinsic self-healing in PUs produced by non-covalent dynamic bonds has been less considered. The majority of these self-healing PUs are synthesized with polyester or polyether polyols, and a few of them have been synthesized with polycarbonate diol polyols; even they may impart excellent chemical and mechanical properties [69,70].

Different self-healing PU gels made with polycarbonates have been reported [71,72]—Scheme 1. Chen et al. have reported improved mechanical properties and excellent self-healing of polymerized hydrogels based on methoxy polyethylene glycol (M_n_ = 5000 Da) and aliphatic polycarbonate—2-methyl-2-benzyloxy carbonyl propylene carbonate (MBC) [71]. MBC monomer acted as rigid segment in the gel network, enhancing the hydrogen bond interactions between carbonyl and hydroxyl groups that were the main responsible of the self-healing at room temperature in 3 h without applying any external stimulus. Similarly, Han et al. have synthesized gels with 5-methyl-5-carboxytrimethylene carbonate and trimethylene carbonate (TMC), and polypropylene glycol (M_n_ = 2000 Da) [72]. These gels showed both shape memory and self-healing (6 h at room temperature without any external stimulus), particularly by increasing the TMC content of the soft segments. The combination of the dynamic network of reversible hydrogen bonds in the gels and their shape memory properties were responsible for the self-healing.

Recently, different self-healing PUs made with polycarbonate diols have been reported [73,74,75,76]—Scheme 1. Zhang et al. synthesized PUs with 5-methyl-5-[(4-methoxy)-benzyloxycarbonyl]-1,3-dioxan-2-one) (MMC), aliphatic polycarbonate, and propylene glycol polyol that showed both shape memory and self-healing at 37 °C in 6 h [73]. The self-healing was made more efficient by decreasing the MMC content (99.3% recovery of its initial mechanical properties) and it was driven by hydrogen bonding and π–π stacking, as well as by the existence of mobile flexible and short chains. On the other hand, Yang et al. synthesized self-healing PUs made with CO_2_-based polycarbonate and amide moieties; the self-healing was ascribed to reversible hydrogen bonds, i.e., hydrogen bonds between amide groups and hydrogen bonds between amide and carbonate groups [74]. In addition, Matějka et al. have synthesized aliphatic PUs by reacting poly(hexamethylene) carbonate diol, hexamethylenediisocyanate, and 1,6-hexanediol chain extender that exhibited self-healing upon heating at 120 °C followed by curing at room temperature [75]. The scratches made on the surface repaired in 1 h, while the fractured bulk PU required 7 h for complete healing. The self-healing was ascribed to molecular diffusion across the interface and chain entanglement. Similarly, Li et al. have synthesized shape-memory and self-healing PUs by reacting polycarbonate diol (M_n_ = 2000 Da), 4,4′-diphenylmethane diisocyanate, and 1,4-butanediol; they exhibited self-healing at 80 °C for 40 s and the extent of self-healing depended on the hard segments content [76]. The self-healing was ascribed to the existence of hydrogen bonds between the hard and the soft segments, and to dipole–dipole interactions between the hard segments.

Despite the advances achieved in developing self-healing PUs, most of them require the application of temperature, or the self-healing time is too long. Therefore, it remains a challenge to develop PUs with fast and controlled healing, particularly rapid self-healing at room temperature without applying external stimuli [52]. Patent EP3103846A reported a PU made with polycarbonate diol polyol that showed rapid self-healing at room temperature without any external stimulus [77]. However, the mechanism by which self-healing in this PU occurs has not been disclosed yet. To understand its self-healing mechanism, in this study, a comparison of the properties of two similarly synthesized PUs with polycarbonate diol polyol (which shows self-healing at room temperature) and polyester polyol (which does not exhibit-self-healing at room temperature) was carried out. Our hypothesis is that the chemical nature of the polyol determines the intrinsic self-healing of the PU at room temperature. In order to confirm our hypothesis, the differences in the structures of the two PUs were addressed by using different experimental techniques, and a mechanism of self-healing based on dynamic non-covalent exchange induced by interactions between the polycarbonate soft segments was proposed.

## 2. Materials and Methods

### 2.1. Materials

Different raw materials were used in the synthesis of the polyurethanes (PUs):

Isocyanate: 4,4′ methylene bis(cyclohexyl) isocyanate (HMDI) (purity 90%). It was supplied by Sigma Aldrich Co. (St. Luis, MO, USA).

Chain extender: 1,4-butanediol (BD) (purity 99%). It was supplied by Panreac Applichem^®^ (Darmstadt, Germany).

Polyols: Polycarbonate of 1,6-hexanediol polyol (CD) (molecular weight = 1000 Da) supplied by UBE Chemical Europe S.A. (Castellón, Spain), and polyadipate of 1,6-hexanediol polyol (PE) (molecular weight of 1000 Da) supplied by Synthesia (Barcelona, Spain).

### 2.2. Methods

#### 2.2.1. Synthesis of the Polyurethanes (PUs)

Two PUs were similarly synthesized with two polyols of different chemical nature and similar molecular weight (1000 Da). The one-shot method was used. An NCO/OH ratio of 1.1 was set. The required amounts of polyol (3.9062 g) and 1,4-butanediol (0.035 g) were placed in a 60 mL polypropylene bottle heated at 80 °C, and the mixture was stirred in double centrifuge SpeedMixer DAC 150.1 FVZ-K equipment (FlackTek Inc., Landrum, SC, USA) at 2400 rpm for 1 min. The mixture was placed in an oven at 80 °C for 10 min. Then, the required amount of HMDI (isocyanate) (1.0938 g) was added to the mixture and stirred again in double centrifuge SpeedMixer equipment at 2400 rpm for 1 min. Afterwards, the PUs were cured in an oven for 7.5 h by following different consecutive stages: 30 min at 50 °C; 30 min at 60 °C; 30 min at 70 °C; and 6 h at 80 °C. After 24 h at room temperature, the PUs were annealed at 85 °C for 1 h. Three PU batches were synthesized, and they were fully reproducible (the amount of each PU batch was 5 g).

**Figure 1:**
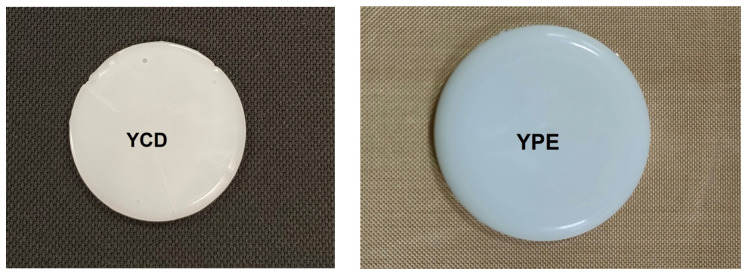
Appearance of the PUs. YCD—PU synthesized with CD polyol; YPE—PU synthesized with PE polyol.

The nomenclature of the two PUs was “YCD”—PU synthesized with CD polyol—and “YPE”—PU synthesized with PE polyol. Figure 1 shows the appearance of the two PUs.

The hard segments content (*HS*) of each PU was calculated by using Equation (1):

(1) $$HS\left(\mathrm{wt}.\%\right)=\frac{W_{iso}+W_{BD}}{W_{iso}+W_{BD}+W_{polyol}}\times 100$$

where *W_iso_* is the weight of isocyanate, *W_BD_* is the weight of chain extender, and *W_polyol_* is the weight of polyol.

The hard segments contents of the PUs were 22 wt.% (PU synthesized with CD polyol) and 23 wt.% (PU synthesized with PE polyol).

#### 2.2.2. Experimental Techniques

**Figure 2:**
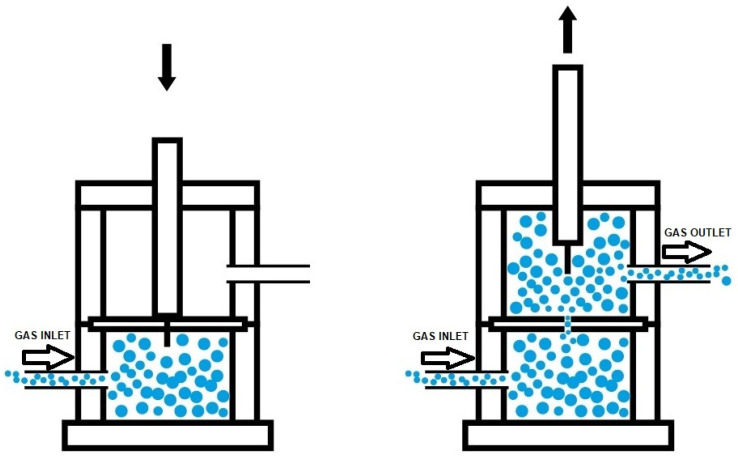
Diagram of the principle of measurement of self-healing and kinetics of self-healing at 20 °C in PUs. The arrows indicate the gas inlet and outlet of the hermetically closed chamber, and the solid blue circles represent the gas bubbles inside the chamber.

Self-healing assessment. The quantitative self-healing at 20 °C of the PUs was determined in the equipment described elsewhere [78]. A cylindrical PU piece of 19 mm diameter and 3 mm thick was placed inside a thermostatic hermetic chamber (Figure 2). The PU piece inside the chamber was fully pierced with a 1 mm diameter needle, and, when the needle was withdrawn, nitrogen gas flowed throughout the hole from bottom to top inside the chamber. The variation in the gas flow was continuously monitored over time until the flow in the gas outlet stopped. The time between the piercing of the PU and the absence of flow in the gas outlet was the self-healing time at 20 °C. Furthermore, the variation in the gas flow through the crack made in the PU over time allowed the determination of the kinetics of self-healing at 20 °C. Three punctures in different locations of each PU were made, and the self-healing results were averaged.

Tack measurement. The tack of the PUs was measured at 20 °C by using the probe tack test. A flat cylindrical probe of 3 mm diameter was brought into contact with the PU surface, a force of 5 N was applied for 1 s, and then the probe was separated at a constant rate of 10 mm/s. As many replicates as necessary were performed to obtain at least 3 concordant values.

Gel permeation chromatography (GPC). GPC was used to determine the molecular weights of the PUs. The experimental setup was standard (pump, injector, column, detector) and consisted of GPC equipment provided with Waters Styragel HR5 and HR3 columns (Waters, Milford, MA, USA), a Waters 1515 isocratic high-performance liquid chromatography pump, and a Waters 2414 refractive index detector. Two mg solid PU was dissolved in HPLC-grade tetrahydrofuran (THF) and the solutions were eluted at a flow rate of 1 mL/min in HPLC-grade THF. Polystyrene standards were used for calibration. Two experiments per sample were measured and averaged.

Infrared spectroscopy in attenuated total reflectance mode (ATR-IR spectroscopy). ATR-IR spectroscopy was used to assess the chemical composition and structure of the polyols and the PUs. An Alpha spectrometer (Bruker Optik GmbH, Ettlinger, Germany) provided with a germanium prism was used, and 60 scans were performed with a resolution of 4 cm^−1^.

Differential scanning calorimetry (DSC). DSC was used to assess the structure and thermal properties of the PUs. Three consecutive thermal runs under a nitrogen atmosphere (flow rate: 100 mL/min) were performed in DSC Q100 equipment (TA Instruments, New Castle, DE, USA): (i) heating from −80 °C to 200 °C (heating rate = 10 °C/min); (ii) cooling from 200 °C to −80 °C (cooling rate = 10 °C/min); and (iii) heating from −80 °C to 250 °C (heating rate = 10 °C/min).

X-ray diffraction (XRD). Wide-angle XRD was used to assess the crystallinity of the polyols and the PUs. Bruker D8-Advance equipment (Bruker, Etlinger, Germany) provided with a nickel filter and a Göebel mirror, a Kritalloflex K 760-80F X-ray generator (3000 W; 20–60 kV; 5–80 mA), and the wavelength of copper (λ = 1.5406 Å) were used. A scan of 2θ angles from 5° to 90° was performed by varying 0.05° every 3 s.

Thermal gravimetric analysis (TGA). TGA was used to assess the structure and thermal properties of the polyols and the PUs. TGA Q500 equipment (TA Instruments, New Castle, DE, USA) was used and the experiments were carried out under a nitrogen atmosphere (flow rate: 50 mL/min). A 9–10 mg sample was placed in a platinum crucible and heated from 35 °C to 600 °C by using a heating rate of 10 °C/min.

Dynamic mechanical thermal analysis (DMA). DMA was used to assess the viscoelastic properties of the PUs. A TA DMA Q800 instrument (New Castle, Delaware, DE, USA) was used, and the experiments were carried out in the single cantilever mode. Rectangular PU samples with dimensions of 33 mm × 12 mm × 2.5 mm were used; they were heated from –100 °C to 80 °C by using a heating rate of 5 °C/min and a frequency of 1 Hz.

X-ray photoelectron spectroscopy (XPS). XPS was used to assess the chemical composition and chemical species on the PU surfaces. An XPS K-ALPHA instrument (Thermo Fisher Scientific, Waltham, MA, USA) provided with a twin crystal monochromator and hemispherical analyzer was used. A sample spot of 400 µm diameter was analyzed by using aluminum kα radiation (1486.6 eV), a current of 3 mA, and a voltage of 12 kV. Charge compensation was achieved with the system’s flood gun. Survey scans with pass energies of 200 eV were obtained and high-resolution C1s, O1s, and N1s spectra were obtained by using pass energies of 50 eV.

Ethylene glycol contact angle measurements. The wettability on the PU surfaces was quantified by ethylene glycol (purity > 99.0%)—supplied by Merck-Schuchardt, Hohenbrunn, Germany—contact angle measurement at 20 °C. An ILMS goniometer (GBX Instruments, Bourg de Pèage, France) was used and ethylene glycol droplets of 3 μL were placed in different locations on the PU surface and measured 15 s after drop deposition. The contact angles were the average of at least 3 drops placed on different zones of the PU surface with an error of less than ±2°.

Stress–strain test. The mechanical properties of the PUs were assessed by stress–strain tests according to the ASTM D 638 standard [79]. Standardized type 2 dumbbell PU specimens were prepared and they were tested in a Zwick/Roell Z005 universal testing machine (Barcelona, Spain) by using a cross-head speed of 100 mm/min. Smooth pneumatic jaws were used. Three replicates for each PU were obtained and the results were averaged.

## 3. Results and Discussion

### 3.1. Assessment of the Self-Healing at 20 °C of the PUs

**Figure 3:**
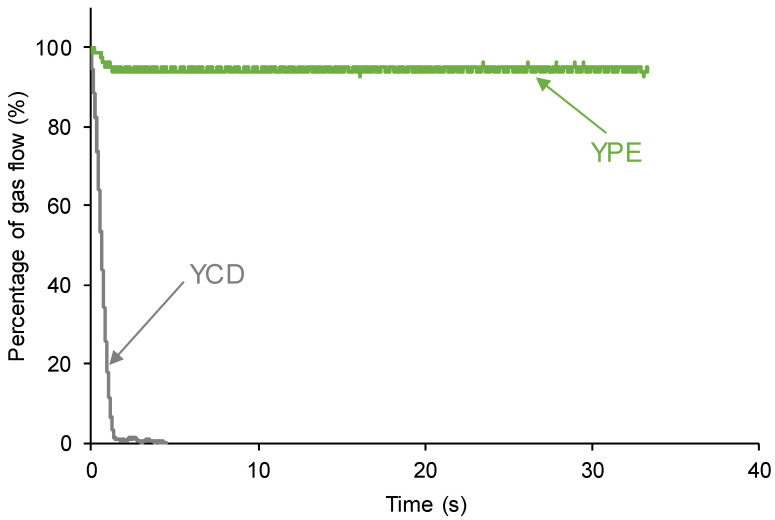
Kinetics of self-healing at room temperature of the PUs.

The self-healing of YCD and YPE polyurethanes was measured in the equipment displayed in Figure 2. YCD shows a quick self-healing at 20 °C (1.4 s), whereas YPE does not show self-healing (Figure 3).

**Figure 4:**
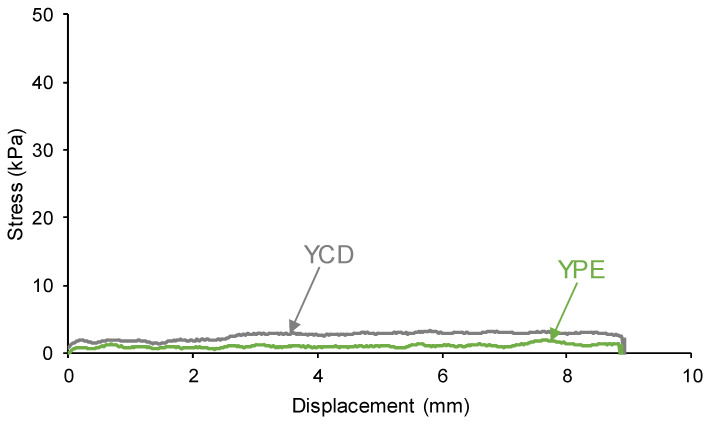
Variation in the stress at 20 °C as a function of the displacement for the PUs. Probe tack test.

Some PUs show tack at room temperature, i.e., the polymeric chains move under the application of mild stresses [80]. Considering that the self-healing of YCD could be due to the existence of tack, the tack of the PUs was assessed by the probe tack test. Figure 4 shows that none of the PUs exhibit tack (i.e., no maximum in the stress–displacement curves was found), and the stress was always lower than 3 kPa. Therefore, the self-healing of YCD cannot be ascribed to the existence of tack.

### 3.2. Structural Characterization of the PUs

YCD and YPE polyurethanes were similarly synthesized, and their hard segments contents are also similar (22–23 wt.%); the only difference between them is the polyol, i.e., polycarbonate diol—CD—or polyester—PE—polyol. Considering that the linear polymers may have displayed self-healing properties via intensive segmental motion, the similarly low hard segments contents in both YCD and YPE would produce similar segmental motion. However, only YCD shows self-healing. The segmental motion of the PUs is tightly related to their molecular weights. The molecular weights of YCD and YPE are moderate (Table 1) as compared to those of other self-healing PUs synthesized with polycarbonate diol polyols [59,74], so they should exhibit good segmental mobility. On the other hand, YCD shows higher molecular weight and polydispersity than YPE, and the M_z_ value is significantly higher in YCD. Consequently, YPE shows a narrower molecular weight distribution than YCD. Therefore, the self-healing of YCD cannot be ascribed only to intensive segmental motion.

Because a short diol, a cycloaliphatic isocyanate, and a polycarbonate diol or a polyester polyol were used in the synthesis of YCD and YPE, the existence of dynamic covalent bonds for explaining the self-healing at 20 °C of YCD can be discarded. Furthermore, considering that the hard segments content in YCD is quite low (22 wt.%), the contribution of hydrogen bonds between urethane, urea, and urethane–urea groups cannot be expected to be sufficient to justify the existence of self-healing at 20 °C. It should be noted that, although only diols were used in the synthesis of the PUs, urea groups should have been formed during the cure in the oven. It is our hypothesis that the self-healing at 20 °C of YCD must be related to the interactions between the polycarbonate soft segments, and that the chemical nature of the polyol determines the self-healing ability of the PUs.

**Figure 5:**
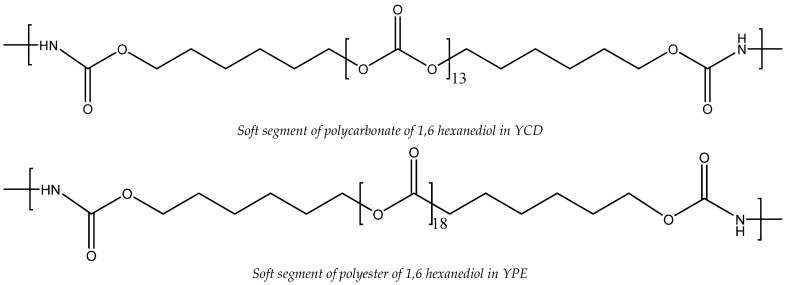
Chemical structure of the soft segments in the PUs.

**Figure 6:**
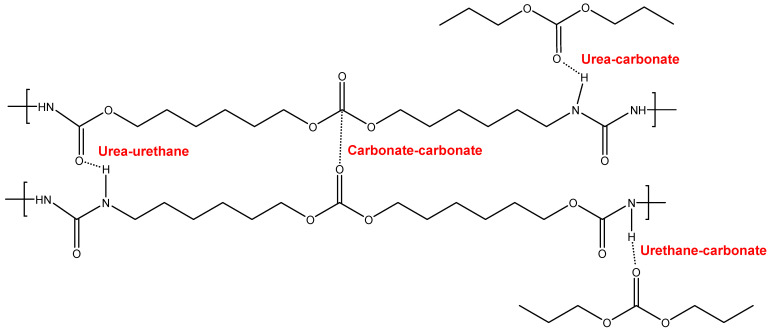
Potential interactions between polar groups in the soft segments of YCD.

**Figure 7:**
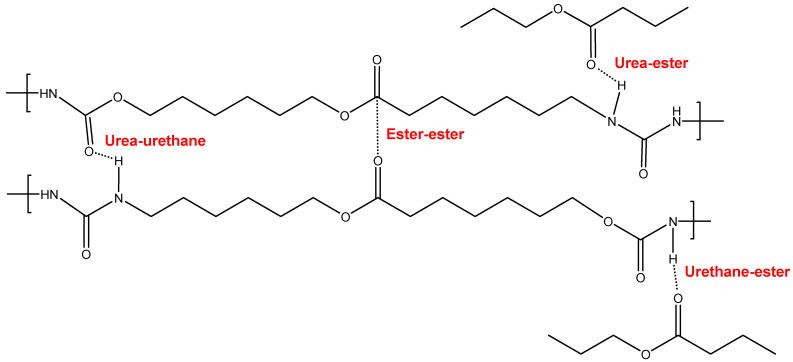
Potential interactions between polar groups in the soft segments of YPE.

Figure 5 depicts a scheme of the polycarbonate and polyester soft segments in the PUs. The figure shows that 13 carbonate groups exist in the soft segments in YCD, and 18 ester groups are present in YPE. Figure 6 and Figure 7 show the schemes of the potential interactions between polar groups in YCD and YPE. Four potential interactions can be anticipated in YCD and YPE: (i) urea–urethane; (ii) urea–ester/urea–carbonate; (iii) urethane–ester/urethane–carbonate; and (iv) ester–ester/carbonate–carbonate. The main difference between YCD and YPE should be in the distinct strength of those interactions. It can be expected that the interactions of the carbonate groups between themselves and with urethane and urea groups in YCD should be stronger than the ones of the ester groups between themselves and with urethane and urea groups in YPE. At the same time, due to the lower number of carbonate groups than ester groups in the soft segments of the PUs, the number of interactions of the carbonate groups between themselves and with urethane and urea groups in YCD will have been lower than the ones of the ester groups between themselves and with urethane and urea groups in YPE.

In this study, the interactions between the polymeric chains in YCD and YPE, and their structural characterization, have been assessed by ATR-IR spectroscopy, DSC, X-ray diffraction, TGA, and DMA.

**Figure 8:**
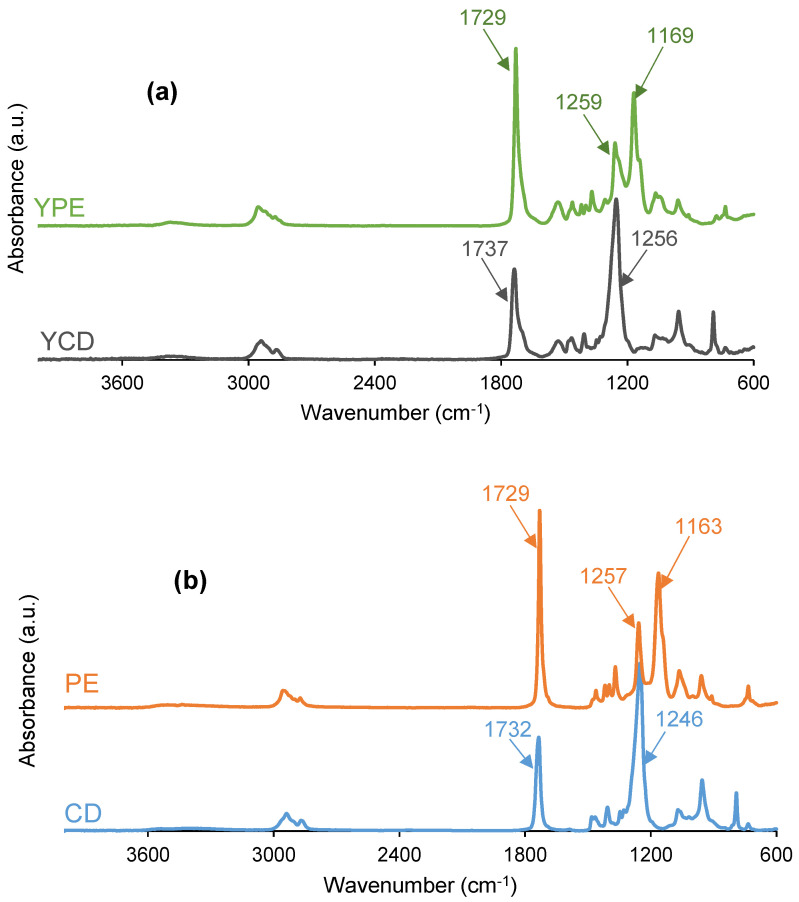
ATR-IR spectra of the PUs (**a**) and the polyols (**b**).

Figure 8 shows the ATR-IR spectra of YCD and YPE. The ATR-IR spectra of both PUs show the same absorption bands, and they differ in the wavenumber of the OCC band of the soft segments (1256 cm^−1^ in YCD, and 1169 and 1259 cm^−1^ in YPE). The main absorption bands of the PUs correspond to the hard segments—N-H stretching at 3356–3364 cm^−1^, C=O stretching due to urethane and urea at 1729–1737 cm^−1^—and the soft segments—C-H stretching at 2938–2954 and 2867–2875 cm^−1^, C-H bending at 1465 and 1346–1370 cm^−1^, and C-O stretching at 900–1256 cm^−1^. The number of ester groups in the soft segments of YPE is higher than the one of carbonate groups in the soft segments of YCD (Figure 5), and, therefore, the intensity of the C=O stretching band is lower in YCD than in YPE. In fact, the ratio of the intensities of the C=O band with respect to that of the OCC band is lower (0.57) in the ATR-IR spectrum of YCD than in the one of YPE (1.35). According to Figure 8, the ratio of the intensities of the C=O band with respect to that of the OCC band in the polyols (CD and PE) is somewhat similar to the ones of the respective PUs, i.e., 0.56 in CD and 1.46 in PE. Therefore, the major differences in the ATR-IR spectra of the PUs are due to the soft segments.

**Figure 9:**
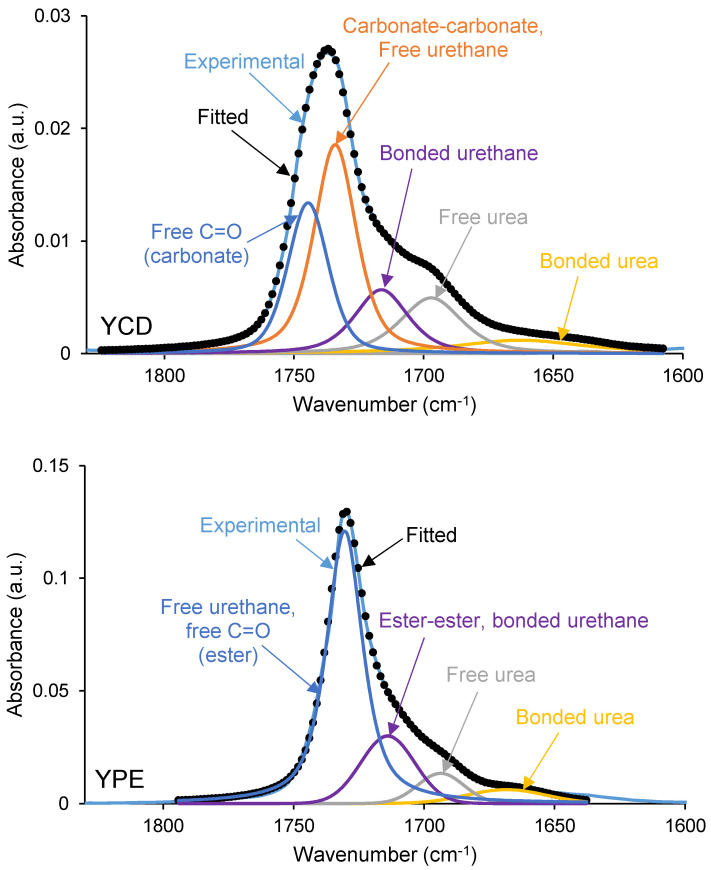
Curve fitting of the carbonyl stretching region of the ATR-IR spectra of the PUs.

The interactions between the polar groups of the PUs were evidenced by curve fitting of the carbonyl stretching region of the ATR-IR spectra. For curve fitting, a Gaussian function was used (Figure 9). The wavenumber of each C=O contribution was assigned according to the previous literature: 1750 cm^−1^—free C=O of carbonate; 1736 cm^−1^—carbonyl–carbonyl interactions in the soft segments; 1730 cm^−1^—free urethane; 1711 cm^−1^—hydrogen-bonded carbonyl groups in the hard and soft segments; 1699 cm^−1^—free urea; and 1660 cm^−1^—hydrogen-bonded urea [81,82,83].

The curve fitting of the C=O stretching region of the ATR-IR spectrum of YCD (Figure 9) shows five contributions: 24% free carbonate groups at 1742 cm^−1^, 38% free urethane and carbonate–carbonate interactions at 1730 cm^−1^, 14% hydrogen-bonded urethane at 1716 cm^−1^, 15% free urea at 1692 cm^−1^, and 9% hydrogen-bonded urea at 1653 cm^−1^ (Table 2). Thus, YCD shows important contributions of free carbonate groups and carbonate–carbonate interactions. On the other hand, the curve fitting of the carbonyl region of YPE (Figure 9, Table 2) shows 56% free ester and free urethane groups at 1732 cm^−1^, 26% hydrogen-bonded urethane at 1719 cm^−1^, 10% free urea at 1695 cm^−1^, and 8% hydrogen-bonded urea at 1667 cm^−1^. The percentages of free and hydrogen-bonded urethane species are higher in YPE than in YCD, whereas the percentage of free urea is higher in YCD (Table 2).

The chemical differences between YCD and YPE rely in the lower percentage of urethane species, the higher percentage of free urea, and the existence of free carbonate groups in YCD with respect to YPE. In fact, a previous study has established that PE polyol had 88% free ester species at 1730 cm^−1^ and 8% bonding by dipole–dipole interactions of C=O groups at 1712 cm^−1^, whereas the CD polyol had 36% free carbonate species at 1741 cm^−1^, and 64% bonding by dipole–dipole interactions of C=O groups at 1730 cm^−1^ [84]. Therefore, there are strong interactions between carbonate groups in the CD polyol and also between the polycarbonate soft segments in YCD.

**Figure 10:**
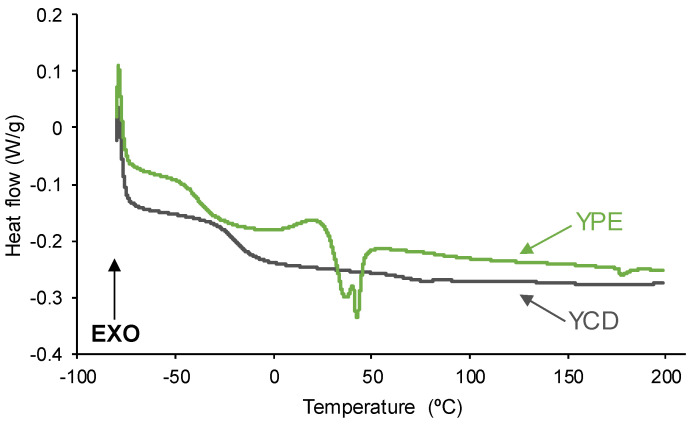
DSC curves of the PUs. First heating run.

The structure of the PUs was also assessed by DSC. The DSC curves of the first heating run of the PUs (Figure 10) show the glass transition temperature (T_g_) of the soft segments at −21 °C (YCD) and −40 °C (YPE); the higher T_g_ value in YCD is due to high polarity of the carbonate groups. The heat capacity at constant pressure (∆c_p_) is higher in YPE (0.35 J/g °C) than in YCD (0.29 J/g °C), indicating stronger interactions between the soft segments in YPE. On the other hand, YPE shows cold crystallization at 21 °C followed by melting of the soft segments at 36–42 °C (melting enthalpy: 7 J/g), whereas YCD exhibits only a small melting at 77 °C (melting enthalpy: 0.1 J/g). Therefore, the movement of the polymeric chains is more restricted in YPE than in YCD; this agrees well with the existence of a higher percentage of urethane groups and less intensive segmental motion of the polymeric chains in YPE.

**Figure 11:**
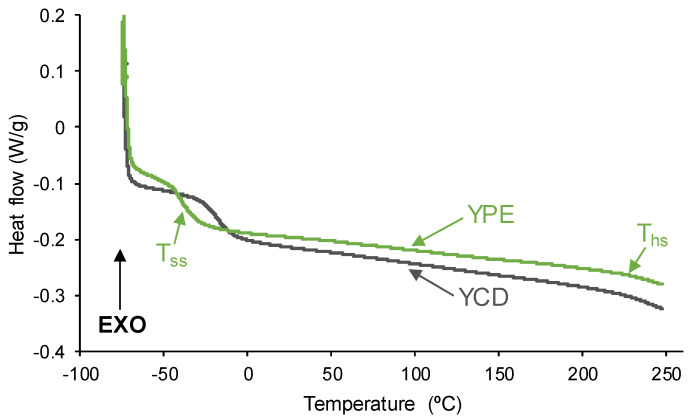
DSC curves of the PUs. Second heating run.

To remove the thermal history of the PUs, after cooling down to −80 °C, a second DSC heating run was carried out (Figure 11). The DSC curves exhibit two glass transitions due to soft segments (T_ss_: −18 °C—YCD -, and −37 °C—YPE) and hard segments (T_hs_: 236 °C—YCD -, and 241 °C—YPE). YCD shows a smaller difference between the T_g_ values of the soft and hard segments, indicating a lower degree of micro-phase separation than in YPE.

**Figure 12:**
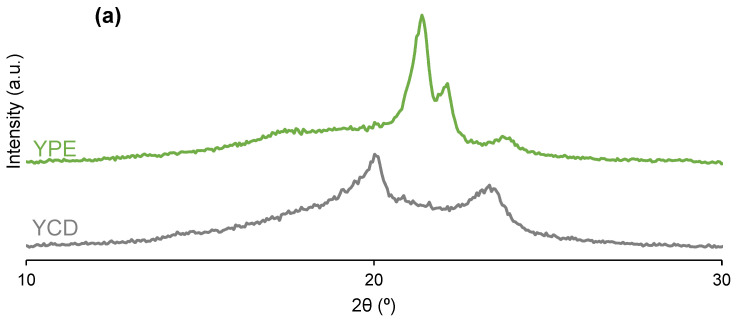

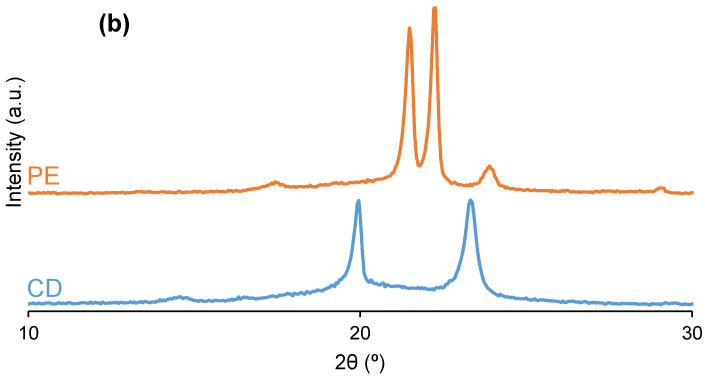
X-ray diffractograms of the PUs (**a**) and polyols (**b**).

Both PUs show crystallinity because they show two main diffraction peaks at 2θ values of 21° and 22° in YPE and at 2θ values of 20° and 23° in YCD (Figure 12). Although the two PUs exhibit crystallinity, their nature is different because of the different 2θ values at which the diffraction peaks appear. Previous studies have reported the existence of an ordered and crystalline structure of the soft segments in PUs synthesized with polycarbonates that was evidenced by two diffraction peaks at 2θ values of 20° and 23° [59,75]. This crystallization assists the micro-phase separation of the soft and hard segments, which can be related to interactions between the soft segments. In fact, the polyols (CD and PE) show diffraction peaks at the same 2θ values as their corresponding PUs (Figure 12), and, therefore, the crystallinity of YCD and YPE should derive from the interactions between the soft segments. The diffraction peaks at 2θ values of 21° and 22° in PE polyol have been ascribed to ester–ester interactions and the ones at 20° and 23° to carbonate–carbonate interactions in CD polyol [84]. On the other hand, the intensities of the diffraction peaks of YPE are higher than the ones of YCD (Table 3) and they are lower than the ones of the corresponding polyols (Table 4), to a greater extent in YPE. This indicates the rupture of some interactions between the polyol chains when they react to obtain the PUs, more noticeably in YPE than in YCD. This is also supported by the fact that the diffraction peaks of the PUs are broader, i.e., less crystalline, than the ones of the corresponding polyols (Figure 12). The higher crystallinity of YPE can be ascribed to its lower molecular weights and polydispersity (Table 1). This may facilitate the alignment and organization into a more ordered structure in YPE than in YCD, and the lower crystallinity of YCD allows more intensive segmental motions of its polymeric chains, which should facilitate self-healing at 20 °C.

**Figure 13:**
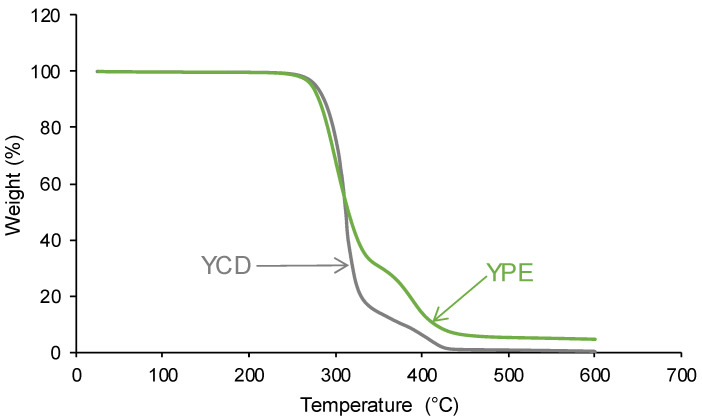
TGA curves of the PUs.

Previous studies have shown that the TGA curves of PUs show differenced thermal degradations of the hard and soft segments [66,80]. Figure 13 shows that the TGA curves of YCD and YPE differ mainly in the thermal decompositions above 300 °C because the decomposition of YPE is less marked due to its lower molecular weight.

**Figure 14:**
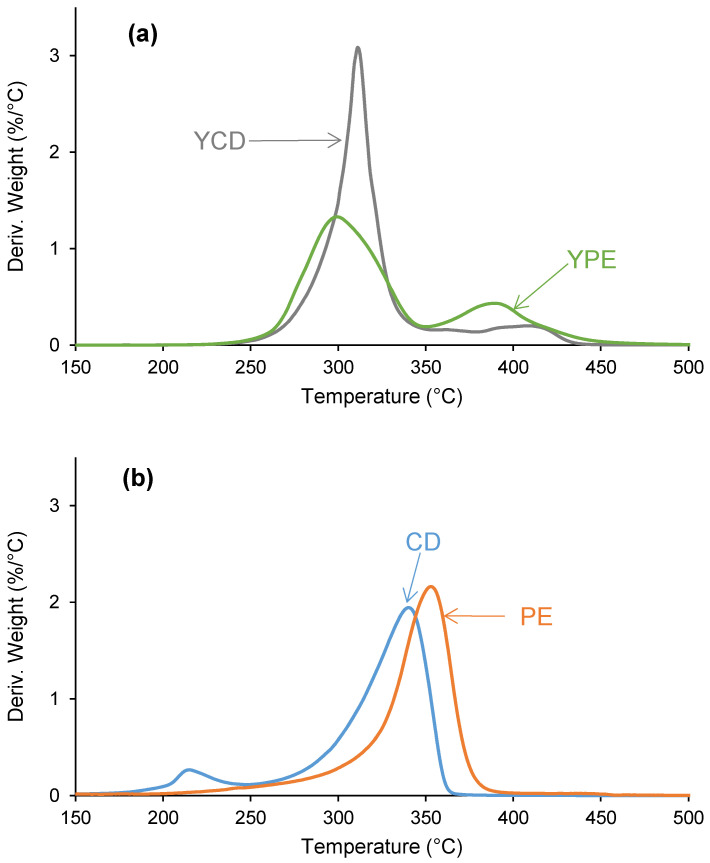
Derivatives of the TGA curves of the PUs (**a**) and the polyols (**b**). TGA experiments.

The differences in the TGA curves of the PUs can be better evidenced in the derivative of the TGA curves (DTGA curves) (Figure 14). The DTGA curve of YCD shows three thermal degradations at 311 °C (likely due to carbonate–carbonate interactions), 340 °C, and 363 °C (likely due to hard segments) with weight losses of 87%, 4%, and 9%, respectively. The thermal degradation at 340 °C in YCD can be ascribed to a mixed phase due to interactions between the carbonyl groups of carbonate and urethane [75,81]. On the other hand, the DTGA curve of YPE shows two thermal degradations at 300 °C (likely due to ester–ester interactions) and 352 °C (likely due to hard segments) with weight losses of 69% and 26%, respectively. The assignment of the thermal degradations is based on the DTGA curves of the polyols (Figure 14) that only show one main thermal degradation at 340 °C and 352 °C, respectively. These thermal degradations have been ascribed to carbonate–carbonate and ester–ester interactions, respectively [84].

The main thermal degradations of YCD and YPE at 311 °C and 300 °C can be ascribed to the interactions between the soft segments, which are more important/stronger in YCD (higher weight loss is obtained). Because the interactions between the soft segments in the PUs are less intensive than in the parent polyols, the temperatures of the thermal decompositions of the PUs at 300–311 °C are lower than the ones of the corresponding polyols (340–352 °C). Whereas the thermal decomposition of the carbonate–carbonate interactions in CD polyol appears at a lower temperature than the one of the ester–ester interactions in PE polyol, the opposite trend is obtained in the PUs. In addition, the thermal degradations at higher temperatures due to the decompositions of the hard segments (urethane and urea) show higher weight losses in YPE because, according to the curve fitting of the carbonyl stretching of the ATR-IR spectra, they are more abundant in YPE than in YCD. In both PUs, the most intensive interactions between the polymeric chains are produced between the soft segments, and YPE shows stronger interactions between hard segments that will favor the micro-phase separation and will limit the movement of its chains. These results agree well with the experimental evidence provided by DSC.

**Figure 15:**
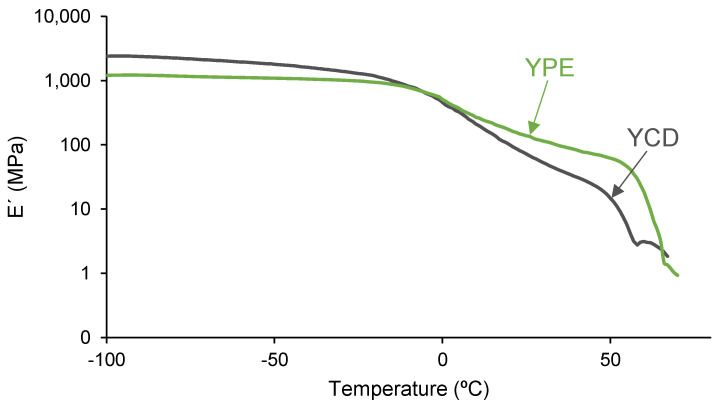
Variation in the storage (E′) modulus as a function of the temperature for PUs. DMA experiments.

**Figure 16:**
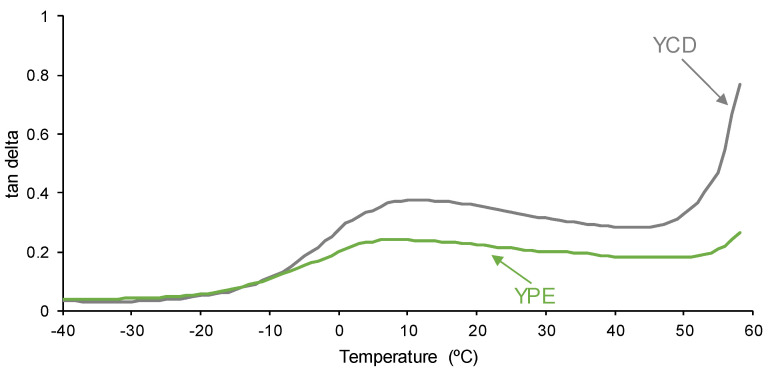
Variation in tan delta as a function of temperature for PUs. DMA experiments.

Because of the structural differences in YCD and YPE, they would show different viscoelastic properties when assessed by DMA. In the glassy region, the storage moduli of YCD are higher than the ones of YPE, but the opposite trend was found in the glass transition and rubbery plateau regions (Figure 15). Once the glass transition was reached, the interactions between the polymeric chains of YCD became significantly weaker than in YPE, and, thus, the mobility of the polymeric chains was favored in YCD with respect to YPE. The tan delta vs temperature plots of the PUs (Figure 16) show only one structural relaxation at a similar temperature (10 °C for YCD and 9 °C for YPE), and the tan delta values in the maxima appear at 0.38 and 0.24, respectively. The higher tan delta value of the structural relaxation of YCD indicates higher loss moduli than in YPE, i.e., more intensive chain mobility due to fewer interactions between the polymeric chains. This agrees well with the experimental evidence shown by DSC and TGA.

In summary, YCD has a significant number of free carbonate groups and a lower percentage of free and hydrogen-bonded urethane groups than YPE. Furthermore, only YPE shows cold crystallization, and YCD exhibits a lower degree of micro-phase separation and lower crystallinity than YPE. In addition, higher weight losses were obtained in the thermal degradation of the hard segments, and lower tan delta values were found in the structural relaxation of YPE than in YCD. All these findings evidence the existence of more mobile polymeric chains in YCD as well as the existence of a significant number of interactions between the polycarbonate soft segments; these two features should favor self-healing at 20 °C in YCD.

### 3.3. Surface Properties of the PUs

The structural differences between YCD and YPE are also evidenced by their surface properties. XPS experiments show that both PU surfaces contain 75 at.% carbon, and they differ in the atomic percentages of oxygen (24.6 at.% on the YCD surface and 23 at.% on the YPE surface) and nitrogen (0.4 at.% on the YCD surface and 2 at.% on the YPE surface). Therefore, the YCD surface is more enriched in oxygen (likely due to higher atomic oxygen content in carbonate groups than in ester groups) and less enriched in nitrogen than the YPE surface.

The curve fittings of the high-resolution N1s photopeaks of the two PU surfaces show one contribution only at a binding energy of 400.0 eV due to the -NH-COO- species of urethane; the percentage of this species is higher in YPE due to its higher urethane content.

**Figure 17:**
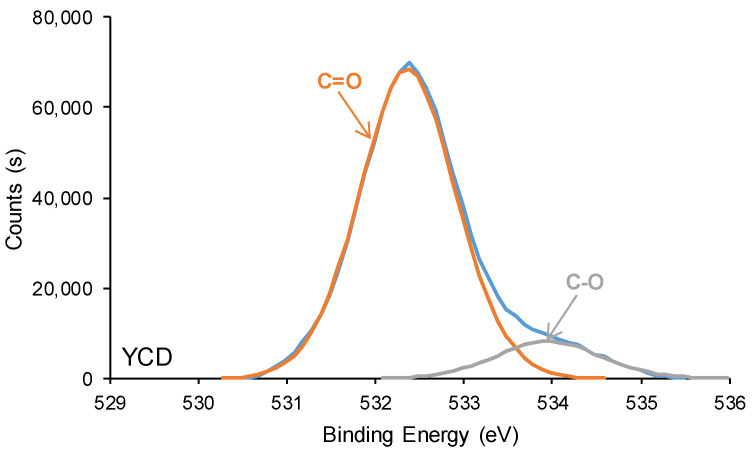

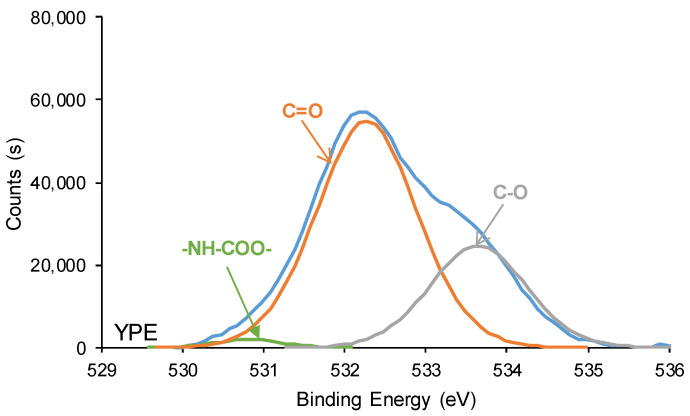
O1s photopeaks of PU surfaces. XPS experiments.

The curve fitting of the high-resolution O1s photopeak of YPE shows a 1 at.% -NH-COO- species at a binding energy of 530.8 eV (Figure 17), and both PU surfaces exhibit different percentages of C-O and C=O species. Thus, the C=O content on the YCD surface is significantly higher (88 at.%) than on the YPE surface (69 at.%); both species appear at similar binding energies (532.3–532.4 eV). The differences in the percentages of C=O species on the PU surfaces are due to the presence of free carbonate groups in YCD. Furthermore, a lower C-O content exists on the YCD surface (12 at.%) than on the YPE surface (30 at.%); this species appears at a slightly higher binding energy (534.0 eV) on the YPC surface than on the YPE surface (533.6 eV). Therefore, the XPS experiments on the PU surfaces show the same trends as the ATR-IR spectra of the PUs.

**Figure 18:**
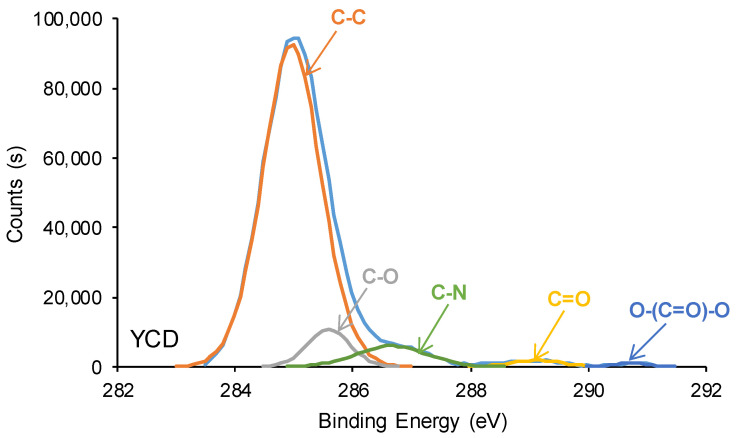

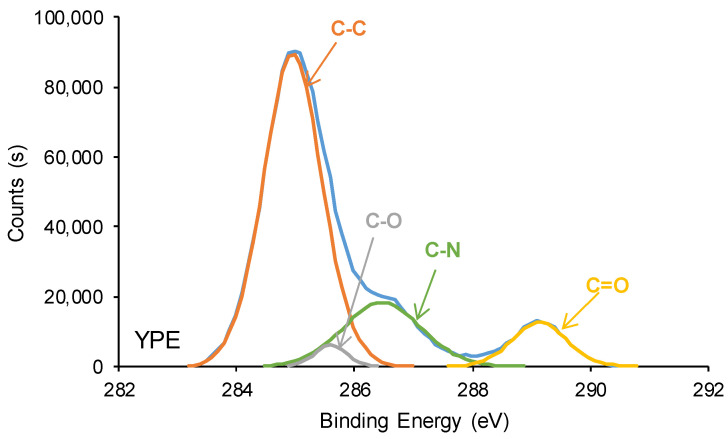
C1s photopeaks of PU surfaces. XPS experiments.

The curve fitting of the high-resolution C1s photopeaks of YCD and YPE surfaces show the existence of C-C (binding energy = 284.9 eV), C-O (binding energy = 285.5–285.6 eV), C-N (binding energy = 286.6–286.9 eV), and C=O (binding energy = 289.1–289.2 eV) species (Figure 18, Table 5); the carbonate groups (binding energy = 290.8 eV) only appear on the YCD surface. The YCD surface shows higher percentages of C-C and C-O and lower percentages of C-N and C=O species than does the YPE surface, due to the existence of higher amounts of urethane and ester species on the YPE surface. On the other hand, the higher amounts of urethane and ester species on the YPE surface with respect to the YCD surface is also evidenced by the significantly lower ethylene glycol contact angle value on the YPE surface (59°) than on the YCD surface (84°). These findings agree well with the evidence provided by ATR-IR, DSC, and TGA. The higher amount of urethane groups on the YPE surface favors the formation of hydrogen bonds and causes the formation of a more ordered phase in the hard segments than on the YCD surface. In turn, the segmental movement of YPE chains would be less favored than in YCD, and this is associated with the absence of self-healing at 20 °C in YPE. Furthermore, the interactions between the polycarbonate soft segments should favor the self-healing of YCD.

### 3.4. Mechanical Properties of the PUs

**Figure 19:**
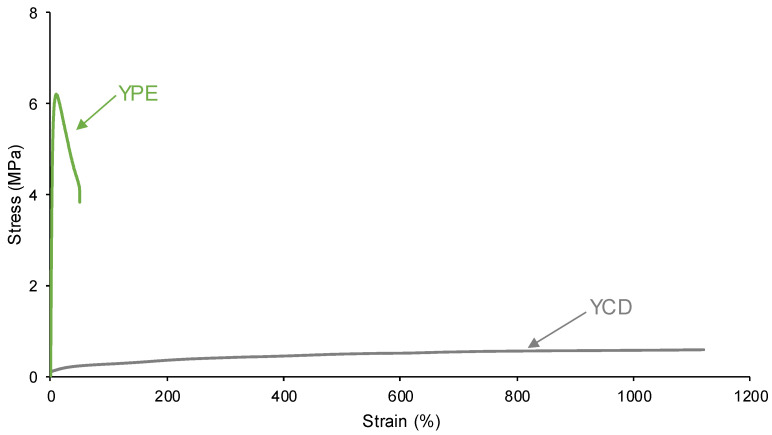
Stress–strain curves of the PUs.

The mechanical properties are important in self-healing PUs, and they are influenced by their structure. The stress–strain curves of YCD and YPE are significantly different (Figure 19). YPE shows the typical stress–strain curve of a stiff thermoplastic material, i.e., somewhat high Young modulus (1.7 MPa), high tensile strength (3.8 MPa), and low elongation-at-break (50%). However, YCD shows the typical stress–strain curve of an elastomeric material, i.e., low tensile strength (0.6 MPa) and elongation-at-break higher than 1120%. Thus, the trends in the mechanical properties of the PUs agree well with the higher amounts of urethane and ester species in YPE than in YCD. On the other hand, due to the absence of cold crystallization and melting of the soft segments in the DSC curve of YCD, its polycarbonate soft segments are quite mobile; this would favor its self-healing at 20 °C.

## 4. Conclusions

Two PUs with similarly low hard segments contents (22–23 wt.%) were synthesized under the same experimental conditions; they only differ in the chemical nature of the polyol (polycarbonate diol, polyester). Whereas the PU made with polycarbonate diol polyol showed intrinsic self-healing at 20 °C, the one made with polyester polyol did not. The self-healing of YCD was not due to the existence of tack, nor to dynamic covalent bonds, nor to extensive hydrogen bonding, but to the interactions between polycarbonate soft segments and intensive segmental mobility of the polymeric chains.

The structural characterization of the PUs showed a significant number of free carbonate groups and a smaller percentage of free and hydrogen-bonded urethane groups in YCD with respect to YPE. Furthermore, YCD exhibited a lower degree of micro-phase separation and lower crystallinity than YPE, and cold crystallization appeared only in YPE. In addition, higher weight losses were obtained in the thermal degradation of the hard segments, and lower tan delta values were found in the structural relaxation, of YPE than in YCD. Whereas the mechanical properties of YPE corresponded to a stiff thermoplastic material, the ones of YCD corresponded to an elastomeric material. All these findings evidenced the existence of mobile polymeric chains in YCD, as well as the existence of a significant number of carbonate groups in the soft segments; these two features favored intrinsic self-healing at 20 °C.

**Figure 20:**
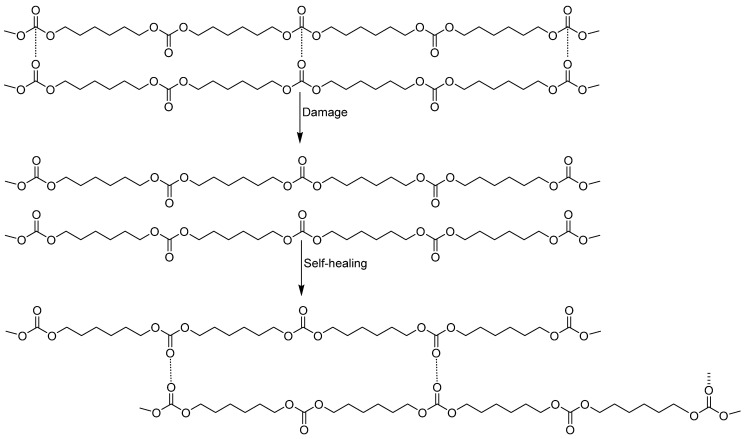
Proposed non-covalent dynamic exchange self-healing mechanism in YCD.

On the basis of the above findings, the proposed mechanism of intrinsic self-healing at 20 °C of YCD was the existence of dynamic non-covalent exchange interactions between mobile polycarbonate soft segments (Figure 20). YCD showed a significant number of free carbonate groups and carbonate–carbonate interactions too. When damage is produced, the rupture of the interactions between polycarbonate soft segments is produced. In a short time, because of the high mobility of the soft segments in YCD and the strong interaction between polycarbonate soft segments, the initially bonded carbonate groups interacted with initially free carbonate groups in the soft segments, the formation of a significant number of new interactions between the polycarbonate soft segments was created, and a fast intrinsic self-healing at 20 °C was produced.

Different self-healing PUs have been proposed for leather coatings, photo-luminescent pigments, flexible electronic components, and biomaterials. Waterborne polyurethane coatings containing disulfide bonds applied on leather require 12 h to self-heal at 60 °C [85], and PUs containing polyimine intended for flexible electronics need 24 h to self-heal at 30 °C [86]. There is little literature on self-healing PUs for biomedical applications. Biocompatible self-healing PUs containing aromatic disulfide bonds exhibit self-healing at 25 °C in 48 h [87], and biocompatible and biodegradable polyoxime PU elastomers show spontaneous self-healing in physiological environments [88].

Considering that YCD polyurethane requires only a short time to self-heal at room temperature and recovers its elastomeric mechanical properties, its potential applications can be mainly directed to the biomedical field, i.e., implantable medical devices and prostheses, and substitutes for sutures and wires in surgery. In our opinion, the extremely fast self-healing and the flexibility of YCD at room temperature are unique properties as compared to previously reported self-healing PUs [71,73,74,75,76,86,87]. Furthermore, the synthesis of YCD is simple and does not require complex stages nor scarcely available functional monomers. In other words, the synthesis of YCD is low-cost and requires only three easily available reagents. However, the poor mechanical properties of YCD limit its application in engineering, and they need improvement.

## Figures and Tables

**Table 1:** Molecular weights of the PUs.

PU	M_n_ (Da)	M_w_ (Da)	M_z_ (Da)	PDI
YCD	19,762	66,779	178,483	3.4
YPE	16,986	40,299	82,150	2.4

**Table 2:** Percentages of different C=O species obtained by curve fitting of the carbonyl stretching region of the ATR-IR spectra of the PUs.

Wavenumber (cm^−1^)	YCD	YPE	Assignment
1653–1667	9	8	Bonded urea
1692–1695	15	10	Free urea
1716–1719	14	26	Ester–ester, bonded urethane
1726–1732	38	56	Carbonate–carbonate, free C=O (ester), free urethane
1742	24	-	free C=O (carbonate)

**Table 3:** Intensities and 2θ values of the main diffraction peaks of the PUs.

PU	2θ (°)	Intensity (a.u.)	2θ (°)	Intensity (a.u.)	2θ (°)	Intensity (a.u.)	2θ (°)	Intensity (a.u.)	2θ (°)	Intensity (a.u.)
YCD	20	3186	-	-	-	-	23	1677	-	-
YPE	-	-	21	4182	22	2092	-	-	24	859

**Table 4:** Intensities and 2θ values of the main diffraction peaks of the polyols.

Polyol	2θ (°)	Intensity (a.u.)	2θ (°)	Intensity (a.u.)	2θ (°)	Intensity (a.u.)	2θ (°)	Intensity (a.u.)	2θ (°)	Intensity (a.u.)
CD	20	3825	-	-	-	-	23	3891	-	-
PE	-	-	21	6042	22	6759	-	-	24	1035

**Table 5:** Chemical species and binding energies (B.E.) on the PU surfaces. C1s photopeak. XPS experiments.

Species	Percentage (at.%)	
	YCD	YPE
C-C, C-H (B.E. = 284.9 eV)	83	69
C-O (B.E. = 285.5–285.6 eV)	7	3
C-N (B.E. = 286.6–286.9 eV)	7	19
C=O (B.E. = 289.1–289.2 eV)	2	9
O-(C=O)-O (B.E. = 290.8 eV)	1	-

## Data Availability

Data are contained within the article.

## References

[1] Zhang M.Q., Rong M.Z. (2022). Extrinsic and Intrinsic Approaches to Self-Healing Polymers and Polymer Composites.

[2] Wen N., Song T., Ji Z., Jiang D., Wu Z., Wang Y., Guo Z. (2021). Recent advancements in self-healing materials: Mechanicals, performances and features. React. Funct. Polym..

[3] Adzima B.J., Kloxin C.J., Bowman C.N. (2010). Externally triggered healing of a thermoreversible covalent network via self-limited hysteresis heating. Adv. Mater..

[4] Yoshie N., Watanabe M., Araki H., Ishida K. (2010). Thermo-responsive mending of polymers crosslinked by thermally reversible covalent bond: Polymers from bisfuranic terminated poly(ethylene adipate) and tris-maleimide. Polym. Degrad. Stab..

[5] Fairbanks B.D., Singh S.P., Bowman C.N., Anseth K.S. (2011). Photodegradable, photoadaptable hydrogels via radical-mediated disulfide fragmentation reaction. Macromolecules.

[6] Chang K., Jia H., Gu S.Y. (2019). A transparent, highly stretchable, self-healing polyurethane based on disulfide bonds. Eur. Polym. J..

[7] Xu Y., Chen D. (2017). Self-healing polyurethane/attapulgite nanocomposites based on disulfide bonds and shape memory effect. Mater. Chem. Phys..

[8] Wang Z., Xie C., Yu C., Fei G., Wang Z., Xia H. (2018). A facile strategy for self-healing polyurethanes containing multiple metal–ligand bonds. Macromol. Rapid Commun..

[9] Chen X., Dam M.A., Ono K., Mal A., Shen H., Nutt S.R., Sheran K., Wudl F. (2002). A thermally re-mendable cross-linked polymeric material. Science.

[10] Ehrhardt D., Van Durme K., Jansen J.F., Van Mele B., Van den Brande N. (2020). Self-healing UV-curable polymer network with reversible Diels-Alder bonds for applications in ambient conditions. Polymer.

[11] Rajeev K.K., Nam J., Kim E., Kim Y., Kim T.H. (2020). A self-healable polymer binder for Si anodes based on reversible Diels–Alder chemistry. Electrochim. Acta..

[12] Lü S., Gao C., Xu X., Bai X., Duan H., Gao N., Feng C., Xiong Y., Liu M. (2015). Injectable and self-healing carbohydrate-based hydrogel for cell encapsulation. ACS Appl. Mater. Interfaces.

[13] Chung C.M., Roh Y.S., Cho S.Y., Kim J.G. (2004). Crack healing in polymeric materials via photochemical [2+2] cycloaddition. Chem. Mater..

[14] Zhu M., Jin H., Shao T., Li Y., Liu J., Gan L., Long M. (2020). Polysaccharide-based fast self-healing ion gel based on acylhydrazone and metal coordination bonds. Mater. Des..

[15] Wei Z., Yang J.H., Liu Z.Q., Xu F., Zhou J.X., Zrínyi M., Osada Y., Chen Y.M. (2015). Novel biocompatible polysaccharide-based self-healing hydrogel. Adv. Funct. Mater..

[16] Xiao G., Wang Y., Zhang H., Chen L., Fu S. (2019). Facile strategy to construct a self-healing and biocompatible cellulose nanocomposite hydrogel via reversible acylhydrazone. Carbohydr. Polym..

[17] Qiao L., Liu C., Liu C., Yang L., Zhang M., Liu W., Wang J., Jian X. (2019). Self-healing alginate hydrogel based on dynamic acylhydrazone and multiple hydrogen bonds. J. Mater. Sci..

[18] Amamoto Y., Kamada J., Otsuka H., Takahara A., Matyjaszewski K. (2011). Repeatable photoinduced self-healing of covalently cross-linked polymers through reshuffling of trithiocarbonate units. Angew. Chem. Int. Ed..

[19] Dong P., Cui K., Xu F., Jiang T., Ma Z. (2018). Synthesis of new ionic crosslinked polymer hydrogel combining polystyrene and poly(4-vinyl pyridine) and its self-healing through a reshuffling reaction of the trithiocarbonate moiety under irradiation of ultraviolet light. Polym. Int..

[20] Yoon J.A., Kamada J., Koynov K., Mohin J., Nicolaÿ R., Zhang Y., Balazs A.C., Kowalewski T., Matyjaszewski K. (2012). Self-healing polymer films based on thiol–disulfide exchange reactions and self-healing kinetics measured using atomic force microscopy. Macromolecules.

[21] Canadell J., Goossens H., Klumperman B. (2011). Self-healing materials based on disulfide links. Macromolecules.

[22] Zheng X., Yang H., Sun Y., Zhang Y., Guo Y. (2021). A molecular dynamics simulation on self-healing behavior based on disulfide bond exchange reactions. Polymer.

[23] Liu M., Zhong J., Li Z., Rong J., Yang K., Zhou J., Shen L., Gao F., Huang X., He H. (2020). A high stiffness and self-healable polyurethane based on disulfide bonds and hydrogen bonding. Eur. Polym. J..

[24] Wu H., Liu X., Sheng D., Zhou Y., Xu S., Xie H., Tian X., Sun Y., Shi B., Yang Y. (2021). High performance and near body temperature induced self-healing thermoplastic polyurethane based on dynamic disulfide and hydrogen bonds. Polymer.

[25] Lee S.H., Shin S.R., Lee D.S. (2019). Self-healing of cross-linked PU via dual-dynamic covalent bonds of a Schiff base from cystine and vanillin. Mater. Des..

[26] Imato K., Nishihara M., Kanehara T., Amamoto Y., Takahara A., Otsuka H. (2012). Self-healing of chemical gels cross-linked by diarylbibenzofuranone-based trigger-free dynamic covalent bonds at room temperature. Angew. Chem..

[27] Cordier P., Tournilhac F., Soulié-Ziakovic C., Leibler L. (2008). Self-healing and thermoreversible rubber from supramolecular assembly. Nature.

[28] Zhao D., Feng M., Zhang L., He B., Chen X., Sun J. (2021). Facile synthesis of self-healing and layered sodium alginate/polyacrylamide hydrogel promoted by dynamic hydrogen bond. Carbohydr. Polym..

[29] Jiang L., Liu B., Zhang J. (2009). Novel high-strength thermoplastic starch reinforced by in situ poly(lactic acid) fibrillation. Macromol. Mater. Eng..

[30] Davydovich D., Urban M.W. (2020). Water accelerated self-healing of hydrophobic copolymers. Nat. Commun..

[31] Chen H., Hao B., Ge P., Chen S. (2020). Highly stretchable, self-healing, and 3D printing prefabricatable hydrophobic association hydrogels with the assistance of electrostatic interaction. Polym. Chem..

[32] Li Y., Zhou T., Yu Z., Wang F., Shi D., Ni Z., Chen M. (2020). Effects of surfactant and ionic concentration on properties of dual physical crosslinking self-healing hydrogels by hydrophobic association and ionic interactions. New J. Chem..

[33] Burattini S., Greenland B.W., Merino D.H., Weng W., Seppala J., Colquhoun H.M., Hayes W., Mackay M.E., Hamley I.W., Rowan S.J. (2010). A healable supramolecular polymer blend based on aromatic π−π stacking and hydrogen-bonding interactions. J. Am. Chem. Soc..

[34] Burattini S., Greenland B.W., Hayes W., Mackay M.E., Rowan S.J., Colquhoun H.M. (2011). A supramolecular polymer based on tweezer-type π−π stacking interactions: Molecular design for healability and enhanced toughness. Chem. Mater..

[35] Xu Z., Peng J., Yan N., Yu H., Zhang S., Liu K., Fang Y. (2013). Simple design but marvelous performances: Molecular gels of superior strength and self-healing properties. Soft Matter..

[36] Burnworth M., Tang L., Kumpfer J.R., Duncan A.J., Beyer F.L., Fiore G.L., Rowan S.J., Weder C. (2011). Optically healable supramolecular polymers. Nature..

[37] Li Z., Shan Y., Wang X., Li H., Yang K., Cui Y. (2020). Self-healing flexible sensor based on metal-ligand coordination. J. Chem. Eng..

[38] Liu Y., Yuan J., Zhang K., Guo K., Yuan L., Wu Y., Gao C. (2020). A novel type of self-healing silicone elastomers with reversible cross-linked network based on the disulfide, hydrogen and metal-ligand bonds. Prog. Org. Coat..

[39] Liu J., Liu Y., Wang Y., Zhu J., Yu J., Hu Z. (2017). Disulfide bonds and metal-ligand co-crosslinked network with improved mechanical and self-healing properties. Mater. Today Commun..

[40] Wang Q., Mynar J.L., Yoshida M., Lee E., Lee M., Okuro K., Kinbara K., Aida T. (2010). High-water-content mouldable hydrogels by mixing clay and a dendritic molecular binder. Nature.

[41] Peng Y., Yang Y., Wu Q., Wang S., Huang G., Wu J. (2018). Strong and tough self-healing elastomers enabled by dual reversible networks formed by ionic interactions and dynamic covalent bonds. Polymer.

[42] Tian X., Yang P., Yi Y., Liu P., Wang T., Shu C., Qu L., Tang W., Zhang Y., Li M. (2020). Self-healing and high stretchable polymer electrolytes based on ionic bonds with high conductivity for lithium batteries. J. Power Sources.

[43] Reisch A., Roger E., Phoeung T., Antheaume C., Orthlieb C., Boulmedais F., Lavalle P., Schlenoff J.B., Frisch B., Schaaf P. (2014). On the benefits of rubbing salt in the cut: Self-healing of saloplastic PAA/PAH compact polyelectrolyte complexes. Adv. Mater..

[44] Yang Y., Ding X., Urban M.W. (2015). Chemical and physical aspects of self-healing materials. Prog. Polym. Sci..

[45] Menon A.V., Madras G., Bose S. (2019). The journey of self-healing and shape memory polyurethanes from bench to translational research. Polym. Chem..

[46] Eyring H. (1936). Viscosity, plasticity, and diffusion as examples of absolute reaction rates. J. Chem. Phys..

[47] Flory P.J., Krigbaum W.R. (1950). Statistical mechanics of dilute polymer solutions. II. J. Chem. Phys..

[48] Simha R., Boyer R.F. (1962). On a general relation involving the glass temperature and coefficients of expansion of polymers. J. Chem. Phys..

[49] Grande A.M., Bijleveld J.C., Garcia S.J., Van Der Zwaag S. (2016). A combined fracture mechanical–rheological study to separate the contributions of hydrogen bonds and disulphide linkages to the healing of poly(urea-urethane) networks. Polymer.

[50] Eschweiler N., Keul H., Millaruelo M., Weberskirch R., Moeller M. (2014). Synthesis of α, ω-isocyanate telechelic polymethacrylate soft segments with activated ester side functionalities and their use for polyurethane synthesis. Polym. Int..

[51] Poljanšek I., Fabjan E., Moderc D., Kukanja D. (2014). The effect of free isocyanate content on properties of one component urethane adhesive. Int. J. Adhes. Adhes..

[52] Qi H.J., Boyce M.C. (2005). Stress–strain behavior of thermoplastic polyurethanes. Mech. Mat..

[53] Yilgör I., Yilgör E., Wilkes G.L. (2015). Critical parameters in designing segmented polyurethanes and their effect on morphology and properties: A comprehensive review. Polymer.

[54] Yu K., Xin A., Feng Z., Lee K.H., Wang Q. (2020). Mechanics of self-healing thermoplastic elastomers. J. Mech. Phys. Solids.

[55] Aguirresarobe R.H., Nevejans S., Reck B., Irusta L., Sardon H., Asua J.M., Ballard N. (2021). Healable and self-healing polyurethanes using dynamic chemistry. Prog. Polym. Sci..

[56] Cho J.W., Kim J.W., Jung Y.C., Goo N.S. (2005). Electroactive shape-memory polyurethane composites incorporating carbon nanotubes. Macromol. Rapid Commun..

[57] Xu Y., Chen D. (2016). A novel self-healing polyurethane based on disulfide bonds. Macromol. Chem. Phys..

[58] Xu Y., Chen D. (2018). Shape memory-assisted self-healing polyurethane inspired by a suture technique. J. Mater. Sci..

[59] Ha Y.M., Kim Y.O., Ahn S., Lee S.K., Lee J.S., Park M., Chung J.W., Jung Y.C. (2019). Robust and stretchable self-healing polyurethane based on polycarbonate diol with different soft-segment molecular weight for flexible devices. Eur. Polym. J..

[60] Sijbesma R.P., Beijer F.H., Brunsveld L., Folmer B.J., Hirschberg J.K., Lange R.F., Lowe J.K.L., Meijer E.W. (1997). Reversible polymers formed from self-complementary monomers using quadruple hydrogen bonding. Science.

[61] Shamiryan D., Abell T., Iacopi F., Maex K. (2004). Low-k dielectric materials. Mater. Today.

[62] Merino D.H., Slark A.T., Colquhoun H.M., Hayes W., Hamley I.W. (2010). Thermo-responsive microphase separated supramolecular polyurethanes. Polym. Chem..

[63] Gooch A., Nedolisa C., Houton K.A., Lindsay C.I., Saiani A., Wilson A.J. (2012). Tunable self-assembled elastomers using triply hydrogen-bonded arrays. Macromolecules.

[64] Feula A., Pethybridge A., Giannakopoulos I., Tang X., Chippindale A., Siviour C.R., Buckley C.P., Hamley I.W., Hayes W. (2015). A thermoreversible supramolecular polyurethane with excellent healing ability at 45 C. Macromolecules.

[65] Li Y., Jin Y., Fan W., Zhou R. (2022). A review on room-temperature self-healing polyurethane: Synthesis, self-healing mechanism and application. J. Leather Sci. Eng..

[66] Ye G., Jiang T. (2021). Preparation and properties of self-healing waterborne polyurethane based on dynamic disulfide bond. Polymers.

[67] Jing T., Heng X., Guifeng X., Li L., Li P., Guo X. (2022). Rapid self-healing and tough polyurethane based on the synergy of multi-level hydrogen and disulfide bonds for healing propellant microcracks. Mater. Chem. Front..

[68] Xu S., Sheng D., Zhou Y., Wu H., Xie H., Tian X., Sun Y., Liu X., Yang Y. (2020). A dual supramolecular crosslinked polyurethane with superior mechanical properties and autonomous self-healing ability. New J. Chem..

[69] García-Pacios V., Costa V., Colera M., Martín-Martínez J.M. (2010). Affect of polydispersity on the properties of waterborne polyurethane dispersions based on polycarbonate polyol. Int. J. Adhes. Adhes..

[70] Kim S.M., Jeon H., Shin S.H., Park S.A., Jegal J., Hwang S.Y., Oh D.X., Park J. (2018). Superior toughness and fast self-healing at room temperature engineered by transparent elastomers. Adv. Mater..

[71] Chen C., Duan N., Chen S., Guo Z., Hu J., Guo J., Chen Z., Yang L. (2020). Synthesis, mechanical properties and self-healing behavior of aliphatic polycarbonate hydrogels based on cooperation hydrogen bonds. J. Mol. Liq..

[72] Han S., Hu Z., Zhang W., Hu J., Yang L. (2022). Flexible segments regulating the gelation behaviours of aliphatic polycarbonate gels with excellent shape memory and self-healing properties. J. Mol. Liq..

[73] Zhang W., Chen S., Chen S., Wang G., Han S., Guo J., Yang L., Hu J. (2023). Physical cross-linked aliphatic polycarbonate with shape-memory and self-healing properties. J. Mol. Liq..

[74] Yang G.W., Zhang Y.Y., Wang Y., Wu G.P., Xu Z.K., Darensbourg D.J. (2018). Construction of autonomic self-healing CO_2_-based polycarbonates via one-pot tandem synthetic strategy. Macromolecules.

[75] Matějka L., Špírková M., Dybal J., Kredatusová J., Hodan J., Zhigunov A., Šlouf M. (2019). Structure evolution during order–disorder transitions in aliphatic polycarbonate based polyurethanes. Self-healing polymer. J. Chem. Eng..

[76] Li S., Zhang J., Chen J., Yao M., Liu X., Jiang Z. (2019). Self-healing polycarbonate-based polyurethane with shape memory behavior. Macromol. Res..

[77] Colera-Llavata M., Costa-Vayá V., Jofre-Reche J.A., Martín-Martínez J.M. (2016). Self-Healing Polyurethane Polymers. Europe Patent.

[78] Paez-Amieva Y., Carpena-Montesinos J., Martín-Martínez J.M. (2023). Innovative device and procedure for in situ quantification of the self-healing ability and kinetics of self-healing of polymeric materials. Polymers.

[79] (2022). Standard Test Method for Tensile Properties of Plastics.

[80] Fuensanta M., Martín-Martínez J.M. (2018). Thermoplastic polyurethane coatings made with mixtures of polyethers of different molecular weights with pressure sensitive adhesion property. Prog. Org. Coat..

[81] Niemczyk A., Piegat A., Olalla Á.S., El Fray M. (2017). New approach to evaluate microphase separation in segmented polyurethanes containing carbonate macrodiol. Eur. Polym. J..

[82] Princi E., Vicini S., Castro K., Capitani D., Proietti N., Mannina L. (2009). On the micro-phase separation in waterborne polyurethanes. Macromol. Chem. Phys..

[83] Fuensanta M., Khoshnood A., Martín-Martínez J.M. (2020). Structure–properties relationship in waterborne poly(urethane-urea)s synthesized with dimethylolpropionic acid (DMPA) internal emulsifier added before, during and after prepolymer formation. Polymers..

[84] Paez-Amieva Y., Martín-Martínez J.M. (2023). Understanding the interactions between soft segments in polyurethanes: Structural synergies in blends of polyester and polycarbonate diol polyols. Polymers.

[85] Liang F., Wang T., Fan H., Xiang J., Chen Y. (2020). A leather coating with self-healing characteristics. J. Leather Sci. Eng..

[86] Lei Y., Wu B., Yuan A., Fu X., Jiang L., Lei J. (2021). Simultaneously self-healing and photoluminescence waterborne polyurethane coatings based on dual dynamic bonds. Prog. Org. Coat..

[87] Eom Y., Kim S.M., Lee M., Jeon H., Park J., Lee E.S., Hwang S.Y., Park J., Oh D.X. (2021). Mechano-responsive hydrogen-bonding array of thermoplastic polyurethane elastomer captures both strength and self-healing. Nat. Commun..

[88] Jiang C., Zhang L., Yang Q., Huang S., Shi H., Long Q., Qian B., Liu Z., Guan Q., Liu M. (2021). Self-healing polyurethane-elastomer with mechanical tunability for multiple biomedical applications in vivo. Nat. Commun..

